# Exploring public perceptions of artificial intelligence in bereavement support: a qualitative study of griefbots

**DOI:** 10.3389/fdgth.2026.1852514

**Published:** 2026-06-02

**Authors:** Jiachun Lu, Meiling Yang, Yingai Cui, Yadan Liu, Thomas Kwok Shing Wong, Yanli Hu

**Affiliations:** 1 School of Nursing, Guangzhou Medical University, Guangzhou, China; 2 School of Nursing, Guangdong Medical University, Zhanjiang, China

**Keywords:** artificial intelligence, bereavement, death, ethics of AI, grief, qualitative research

## Abstract

**Background:**

Artificial intelligence technologies designed to simulate deceased loved ones, often referred to as griefbots, are emerging as a novel form of bereavement support. Although such technologies may offer emotional comfort and a sense of continued connection with the deceased, they also raise complex psychological, ethical, and societal concerns. This study aimed to explore public perceptions of griefbots in bereavement support, with particular attention to perceived benefits, psychological and social risks, ethical concerns, and expectations for responsible governance and institutional integration.

**Methods:**

A descriptive phenomenological approach was employed. Semi-structured interviews were conducted with 25 participants from Guangdong Province, China, and data were analyzed using Colaizzi's seven-step method. Written informed consent was obtained from all participants, and the study received ethics approval from Guangzhou Medical University.

**Results:**

Four themes were identified. Participants perceived griefbots as offering psychosocial benefits, particularly emotional comfort, continued connection with the deceased, and a symbolic means of addressing unresolved loss and regret. At the same time, they expressed concerns about disruption of natural grieving processes, inauthenticity, dependency, psychological harm, social inequality, stigma, and ethical tensions related to data use and posthumous representation. Participants also articulated expectations regarding technology design, use boundaries, governance, and legal safeguards. Overall, perceived acceptability was not organized around simple approval or rejection, but was described as conditional, shaped by context, individual readiness, and perceived vulnerability.

**Conclusion:**

Griefbots were perceived as neither inherently beneficial nor inherently harmful. Instead, their comfort and risk were understood to arise from the same capacity to simulate the continued presence of the deceased. Public perceptions of AI in bereavement support were therefore best understood as conditional and negotiated, underscoring the need for careful governance and responsible integration in bereavement-related contexts.

## 1 Introduction

The loss of a loved one is a universal human experience, often triggering profound psychological and physiological responses collectively referred to as bereavement grief (1). For most individuals, the intensity of such grief naturally diminishes over time, allowing for a gradual return to normal functioning. However, for a significant subset of individuals, grief may evolve into Prolonged Grief Disorder (PGD)—a condition marked by an intense and persistent yearning for the deceased, along with other debilitating symptoms that hinder daily functioning. PGD can have a severe impact on an individual's emotional well-being, leading to prolonged distress and functional impairment (2). Research indicates that approximately 9.8% of individuals who experience natural bereavement develop PGD (3, 4), with this rate rising dramatically to 49% among those who suffer an unnatural or traumatic loss (5), such as through accidents, suicide, or violence. Bereaved parents of only children, as well as individuals who lose loved ones to violence, are particularly vulnerable to developing PGD (6). *This high prevalence underscores the urgent need for more effective interventions. However, current treatment options for PGD remain limited. Although conventional psychological therapies—such as grief counseling and cognitive-behavioral therapy—have demonstrated some efficacy, they are often constrained by issues related to accessibility, timeliness, and individual adaptability. Accordingly, there is an urgent need to explore more diverse and effective adjunctive intervention approaches* (7, 8).

In response to the growing demand for effective grief support, AI-driven technologies have emerged as a promising solution. AI-driven resurrection technologies, including the development of griefbots (AI-generated digital avatars of the deceased), have been created to simulate the presence of lost loved ones. These griefbots use input data such as photographs, voice recordings, and videos to generate digital representations that replicate the deceased's appearance, voice, and personality traits (9). By interacting with these digital versions, bereaved individuals can maintain an emotional connection with their loved ones, offering comfort and closure, and complementing traditional grief management strategies during the mourning process.

With the rapid advancement and proliferation of generative artificial intelligence (AIGC) technologies, the accessibility of griefbot-related applications has significantly improved, leading to a gradual emergence of such services in the market (10, 11). For instance, Zhang reported a surge in demand for customized AI-generated digital human services since 2022, with over 600 orders fulfilled within a single year and pricing ranging from several thousand to tens of thousands of yuan. These cases reflect a growing societal desire for “digital immortality” and emotional continuity with the deceased, highlighting the rising influence of griefbots as a socio-technological phenomenon (12).

As a form of Socially Assistive Robots (SARs), griefbots offer non-contact emotional interaction. They have the potential to alleviate loneliness, sadness, and psychological distress in grieving individuals (13). Studies show that interacting with AI can provide comfort, especially in the early stages of grief (14–16). This interaction helps maintain a “psychological connection” with the deceased, providing transitional support and helping individuals gradually adapt to their loss. Griefbots are particularly beneficial for those who struggle to obtain sufficient support through traditional social networks, such as elderly individuals living alone or families who have lost a child (17).

However, the psychological benefits of griefbots are not without controversy. Critics argue that over-reliance on AI simulations of the deceased may hinder the natural grieving process and create unhealthy attachments, preventing individuals from reintegrating into real-life relationships (18–20). For users, excessive dependence on technology could worsen loneliness and hinder psychological adjustment. Prolonged interaction with a “virtual deceased” may blur the lines between reality and the virtual world, negatively affecting emotional regulation, especially without proper guidance. A tragic case in February 2024, where a 14-year-old boy in the United States ended his life after interacting with the AI chatbot “Garcia,” has raised concerns about the potential for griefbots to escalate psychological crises and induce extreme behavior (21). This underscores the urgent need for risk assessments and usage guidelines.

As griefbots become increasingly commercialized, the market has diversified, with varying prices and product quality. Many griefbot products lack professional psychological or ethical guidance. Some raise concerns about data security and misleading marketing (22–24). More importantly, there is currently no clear legal or ethical regulatory framework for griefbots, leaving the field in a state of “technology ahead of regulation.” This underscores the need for more structured governance to ensure safe and ethical use (25).

Recent research on digital clones of the deceased has begun to address these challenges by linking empirical findings with normative and institutional analysis. Prior work highlights the role of the deceased person's consent in shaping social acceptability (26, 27), alongside concerns related to data use, identity representation, and user protection (19). Institutional approaches further propose differentiated governance based on user type and purpose of use (28), while neuroethical perspectives emphasize mental privacy and protection against exploitation (29). However, less is known about how the public interprets and evaluates these issues in the context of griefbots.

This study aimed to explore public perceptions of griefbots in bereavement support, with particular attention to perceived benefits, psychological and social risks, ethical concerns, and expectations for responsible governance. By examining how members of the public evaluate griefbots in relation to user vulnerability, purposes of use, posthumous representation, and contextual conditions, this study seeks to clarify the conditional structure of acceptability. In doing so, it provides empirical insight that can inform ethical analysis and the institutional design of griefbot governance in digitally mediated care settings.

## 2 Materials and methods

### 2.1 Study design

A descriptive phenomenological approach was employed to explore public perceptions of griefbots.

### 2.2 Participants, recruitment, and sampling

Participants were recruited in Guangdong Province, China, using maximum variation sampling combined with snowball sampling to ensure diversity in age, occupation, and other key demographic characteristics, thereby capturing a broad range of perspectives. Semi-structured interviews were conducted between October 1 and October 14, 2024, using a combination of face-to-face and telephone interviews, depending on participants' availability and preference.

Participants were eligible if they were aged 18 years or older, were able to communicate in Chinese, and had at least a basic understanding of AI technologies used to digitally simulate or “resurrect” deceased individuals. Bereavement experience was not required for inclusion because the study aimed to explore broader public perceptions rather than the experiences of bereaved users only. Prior to the interviews, participants were provided with a brief explanation of griefbots to ensure adequate understanding of the topic. The demographic characteristics of the participants are presented in Table 1.

**Table 1:** Demographic characteristics of participants.

Participant	Gender	Age	Occupation	Marital status	Residence	Per-capita monthly household income, CNY	Bereavement experience	Time since loss
P1	Male	20–29	Nurse	Unmarried	Urban	≥5,000	No	N/A
P2	Male	20–29	Physician	Unmarried	Urban	3,000–3,999	Yes	>12 months
P3	Female	40–49	Nurse	Unmarried	Urban	≥5,000	Yes	>12 months
P4	Female	30–39	Teacher	Married	Urban	≥5,000	Yes	>12 months
P5	Female	40–49	Nurse	Remarried	Urban	≥5,000	Yes	>12 months
P6	Female	40–49	Teacher	Married	Urban	≥5,000	Yes	>12 months
P7	Male	20–29	Programmer	Unmarried	Urban	≥5,000	No	N/A
P8	Female	50–59	Nurse	Married	Urban	≥5,000	Yes	>12 months
P9	Female	20–29	Teacher	Unmarried	Urban	≥5,000	Yes	>12 months
P10	Male	20–29	Other	Unmarried	Rural	≥5,000	Yes	3–6 months
P11	Male	50–59	Teacher	Married	Urban	≥5,000	Yes	>12 months
P12	Male	20–29	Lawyer	Unmarried	Urban	4,000–4,999	Yes	>12 months
P13	Male	30–39	Teacher	Married	Urban	≥5,000	Yes	>12 months
P14	Female	30–39	Teacher	Married	Urban	≥5,000	Yes	>12 months
P15	Male	20–29	Programmer	Unmarried	Rural	4,000–4,999	Yes	>12 months
P16	Female	50–59	Teacher	Divorced	Urban	≥5,000	Yes	>12 months
P17	Female	30–39	Teacher	Married	Rural	≥5,000	Yes	>12 months
P18	Female	20–29	Lawyer	Unmarried	Urban	4,000–4,999	Yes	>12 months
P19	Female	20–29	Physician	Unmarried	Rural	3,000–3,999	Yes	3–6 months
P20	Female	20–29	Physician	Unmarried	Urban	3,000–3,999	Yes	>12 months
P21	Male	30–39	Teacher	Married	Urban	≥5,000	Yes	>12 months
P22	Female	20–29	Lawyer	Unmarried	Urban	4,000–4,999	Yes	>12 months
P23	Female	20–29	Nurse	Unmarried	Rural	3,000–3,999	Yes	>12 months
P24	Male	30–39	Teacher	Married	Urban	≥5,000	Yes	3–6 months
P25	Male	20–29	Nurse	Unmarried	Urban	3,000–3,999	Yes	>12 months

All participants held a bachelor's degree or above. N/A means not applicable.

### 2.3 Interview guide

The interview guide was developed by the research team, reviewed by experts, and refined through pre-interviews with two participants. Feedback from these pre-interviews informed further revisions, and data from these participants were included in the analysis. The final version of the interview guide is presented in Table 2.

**Table 2:** Interview guide.

Guiding Questions
1. Have you heard of AI technologies used to “resurrect” deceased loved ones? What is your opinion on this concept?
2. If it were possible to “resurrect” your lost loved ones through a screen-based simulation where they can interact with you, would you be open to using such a service? Why or why not?
3. What potential benefits do you think the emergence of AI “resurrection” technologies could bring? Please consider both emotional and societal implications.
4. What potential drawbacks or risks do you foresee arising from the development of this industry? How might these risks impact individuals or society as a whole?
5. In your opinion, what improvements are needed for the responsible and healthy development of AI “resurrecting” technologies? What ethical considerations should be prioritized?
6. Do you think it would be appropriate to classify AI technologies that “resurrect” deceased loved ones as medical devices? Why or why not?
7. Would you be willing to use an AI system designed to accompany you or your loved ones in the future (e.g., as a companion or support tool)? What factors would influence your decision?
8. Is there anything else you would like to share regarding the concept of AI “resurrecting” loved ones? Any additional thoughts or concerns?

### 2.4 Data collection and analysis

Interviews were conducted and audio-recorded with participant consent, and the recordings were securely stored. Each participant was assigned a unique identifier to ensure confidentiality. Interviews lasted 45–60 min, with transcriptions completed within one hour and verified for accuracy by two independent reviewers. Data analysis followed Colaizzi's seven-step phenomenological method (30), supported by NVivo 14 software for systematic theme identification.

### 2.5 Credibility

The credibility of the study was enhanced through maximum variation sampling, which ensured a wide range of perspectives. Additionally, the data collection and analysis processes were reviewed by two independent reviewers to ensure rigor. Colaizzi's seven-step method provided a structured framework for data interpretation, and NVivo 14 software supported reliable data management and coding.

### 2.6 Ethical considerations

The study was conducted in accordance with the Declaration of Helsinki, with informed consent, voluntary participation, and strict confidentiality measures. Participation was voluntary, and all participants were informed that they could withdraw from the study at any time without penalty. The study was approved by the Ethics Committee of Guangzhou Medical University (approval No. L202306002).

## 3 Results

The analysis identified four themes and thirteen subthemes that reflected how participants understood and evaluated griefbots (Table 3). Across the interviews, participants did not frame griefbots in simply positive or negative terms, but weighed potential benefits against emotional, ethical, and governance-related concerns.

**Table 3:** Themes and subthemes.

Theme	Subthemes
Perceived Psychosocial Benefits of Griefbots	Emotional Comfort and Continued Connection
	Addressing Unresolved Loss and Regret
Perceived Psychological and Social Risks of Griefbots	Disruption of Natural Grieving Processes and Existential Order
	Perceived Inauthenticity and Identity Distortion
	Risk of Dependency and Impaired Social Functioning
	Potential Psychological Harm and Prolonged Grief
Ethical and Societal Concerns About Griefbots	Social Inequality and Access Barriers
	Social Stigma and Non-Normative Grief Expression
	Ethical Tensions in Data Use and Posthumous Representation
Expectations for the Responsible Integration of Griefbots	Expectations for Technology Design and Functionality
	Expectations for Appropriate Use Boundaries
	Expectations for Governance and Regulatory Oversight
	Institutional and Legal Safeguards for Griefbot Use

More fine-grained categories within Theme 4 are described in the main text.

### 3.1 Perceived psychosocial benefits of griefbots

Participants described griefbots as offering a range of perceived psychosocial benefits, primarily related to emotional regulation and the symbolic continuation of relationships with the deceased. These benefits were reflected in two interrelated sub-themes.

#### 3.1.1 Emotional comfort and continued connection

Participants consistently described griefbots as providing emotional comfort, particularly following sudden or unexpected loss. The technology enabled an ongoing sense of interaction with the deceased, fostering a perceived continuation of the relationship. Participants described this ongoing sense of interaction as emotionally comforting.

“If this technology is used after the sudden death of a loved one… it can help alleviate sorrow, reduce trauma, and support emotional recovery.” (P6)

Beyond immediate grief relief, participants also emphasized the symbolic reassurance provided by griefbots. Some linked this reassurance to feeling less fearful or distressed about death. The perceived persistence of the deceased contributed to reduced grief intensity and mitigated anticipatory death anxiety.

“This technology reassures individuals that even after death, they can maintain a connection, as if their loved one is still present.” (P5)

#### 3.1.2 Addressing unresolved loss and regret

Participants further highlighted the role of griefbots in addressing unresolved loss and lingering regret following bereavement. Death was frequently described as disrupting relational continuity, leaving emotional needs unmet and experiences incomplete. In this context, griefbots were perceived as offering a way to symbolically revisit or continue relationships that had been interrupted by death.

For individuals with limited or fragmented memories of the deceased, griefbots were seen as facilitating a reconstructed sense of connection, particularly in supporting ongoing emotional development.

“If a child has only a vague memory of a deceased parent, this technology could support their emotional development.” (P8)

Participants also described griefbots as enabling imagined forms of closure, including the expression of unspoken thoughts and simulated final interactions.

“This technology helps address regrets, such as being unable to say goodbye or express final words.” (P3)

The same features that made griefbots comforting also laid the groundwork for concerns about dependency and difficulty letting go.

### 3.2 Perceived psychological and social risks of griefbots

Participants reported several psychological and social risks associated with griefbot use, including disruptions to natural grieving processes, perceived inauthenticity, dependency, and potential emotional harm.

#### 3.2.1 Disruption of natural grieving processes and existential order

Some participants conceptualized death as an inherent and natural component of the life course and viewed technological “resurrection” as a disruption of this existential order. From this perspective, the use of griefbots was seen as conflicting with fundamental beliefs about the finality of death and the boundaries between life and non-existence.

“Why would you want to resurrect someone who has already passed away? They are gone; what is the point of bringing them back?” (P4)

In addition, some participants emphasized that accepting death as a natural process, and maintaining internalized memories of the deceased, may reduce the perceived need for technological mediation.

“If we cherish our time together and accept death as natural, the need for AI resurrection would greatly decrease. We can always remember the deceased in our hearts without relying on AI.” (P23)

#### 3.2.2 Perceived inauthenticity and identity distortion

Participants also expressed concerns that griefbots may feel inauthentic, particularly when their responses, appearance, or personality differed from how the deceased was remembered. Such perceived inaccuracies were seen as potentially distorting the identity of the deceased and producing experiences that felt incongruent or unsettling.

“If the AI differs greatly from my memories of my loved one, I might find it ridiculous because it would make me realize it's not truly them.” (P2)

Some participants further questioned whether digitally reconstructed interactions could meaningfully represent the deceased as a person rather than as a data-based simulation. In this sense, griefbots were seen by some as “not truly” the deceased, but rather as a constructed approximation that might undermine the integrity of their memory and identity.

“If we use AI to resurrect the deceased, do you really think this resurrected AI is your loved one? It's just a collection of data.” (P23)

Concerns also centered on the capacity of AI to authentically represent the agency and subjectivity of the deceased. Participants questioned whether simulated responses could meaningfully reflect the individual's intentions, values, or “free will.”

“I’m concerned that AI might not truly represent the deceased's free will. Even if AI attempts to replicate their personality, it may not fully restore them, raising ethical issues like changes to their appearance or manipulation.” (P10)

#### 3.2.3 Risk of dependency and impaired social functioning

Participants expressed concerns about the potential for over-dependence on griefbots, which may impair individuals' ability to function in everyday life. Excessive reliance on simulated interactions was seen as potentially displacing real-world engagement and hindering adaptive coping.

“I might worry that if I become overly dependent on this, it could negatively affect my future life.” (P9)

Participants also highlighted the risk of disrupted family dynamics and impaired social relationships. Immersion in ongoing interactions with a digital representation of the deceased was perceived as limiting individuals' capacity to form new relationships or maintain existing social roles.

“If a person is constantly immersed in interactions with an AI resurrection of their loved one, it could hinder their ability to form new relationships or disrupt the normal functioning of the family.” (P11)

Several participants further noted that such dependency may make it harder for individuals to return to everyday routines and social relationships.

#### 3.2.4 Potential psychological harm and prolonged grief

Participants raised concerns regarding potential psychological harm associated with griefbots, particularly in relation to prolonged grief responses. Some participants worried that this renewed sense of presence could make later separation feel more painful.

“For those who don’t want this, every interaction might be psychological harm.” (P16)

Some participants suggested that, while griefbots may offer temporary comfort, they could make it harder for some individuals to move forward because they remained emotionally focused on the deceased.

“It might keep them trapped in the past or prevent them from moving forward.” (P11)

Participants emphasized that such unintended emotional consequences may be especially pronounced among individuals who are not psychologically prepared to engage with representations of the deceased, raising questions about how such risks should be appropriately managed in practice.

### 3.3 Ethical and societal concerns about griefbots

Participants also raised concerns that extend beyond individual experiences, highlighting broader ethical and societal issues related to the use of griefbots. These concerns encompass questions of equity, social norms, and the governance of emerging technologies.

#### 3.3.1 Social inequality and access barriers

Participants raised concerns that the introduction of griefbot technology may exacerbate existing social inequalities, particularly due to financial barriers limiting accessibility. High costs were perceived as restricting access to more affluent groups, thereby creating inequitable opportunities for benefit.

“This service is currently very expensive, and not everyone can afford it, which could create social inequality.” (P5)

“We also need to think about how to ensure the service remains affordable so that more people can benefit from it.” (P24)

Beyond financial constraints, participants highlighted that unequal access may intersect with social vulnerability, placing certain groups at greater risk of exploitation. Individuals with lower levels of education or digital literacy were perceived as particularly susceptible.

“There may be fraud, as some unscrupulous businesses might exploit this technology to deceive families, especially those who are less educated or more easily tricked.” (P17)

#### 3.3.2 Social stigma and non-normative grief expression

Participants suggested that the use of griefbots may be subject to social stigma, particularly among individuals unfamiliar with or resistant to the technology. However, participants' accounts more directly reflected concerns about how certain forms of grief expression may be socially judged or stigmatized. These forms were often described as prolonged, immersive, or unconventional.

“If people know someone's child has died and they say, ‘I’m going home to be with my child,’ it might seem bizarre or crazy to others.” (P4)

These accounts suggest that the potential stigma surrounding griefbot use may not stem solely from the technology itself, but from broader societal discomfort with non-normative or continuing bonds-oriented grief practices, which are sometimes interpreted through a mental health stigma lens.

#### 3.3.3 Ethical tensions in data use and posthumous representation

Participants expressed significant concerns regarding privacy, data security, and the ethical use of personal information. The potential misuse of sensitive data by AI developers was seen as a key risk, raising questions about confidentiality and accountability.

“I worry that the AI company might misuse the data or violate the privacy and dignity of both the user and the deceased.” (P8)

In addition, participants questioned the ethical legitimacy of reconstructing deceased individuals without explicit consent. Concerns were raised about whether such representations might distort identity or undermine respect for the deceased.

“If I create an AI of my grandfather based on my memories, does this erase his real existence? Is this technology disrespectful to my deceased relative?” (P12)

Taken together, these accounts suggest that participants' views were not defined by simple acceptance or rejection. Rather, the perceived acceptability of griefbots was often described as contingent on family circumstances, cultural background, and individual readiness.

### 3.4 Expectations for the responsible integration of griefbots

Building on these conditional and negotiated views, participants articulated a range of expectations regarding how griefbot technology should be designed, used, and regulated to support its safe and acceptable integration into everyday life. These expectations concerned issues such as design principles, use boundaries, regulatory oversight, and legal safeguards.

#### 3.4.1 Expectations for technology design and functionality

Participants articulated clear expectations regarding how griefbots should be designed to ensure acceptability and meaningful engagement. These expectations focused on alignment with sociocultural values, personalization of user experience, realism in representing the deceased, and the integration of multidisciplinary expertise.

Participants emphasized that griefbots should operate within established legal and cultural frameworks to avoid ethical conflicts.

“We need to strictly review the ‘resurrection’ content to ensure that it aligns with Chinese values and laws.” (P13)

Personalization was also considered essential, with participants expecting griefbots to adapt to users' emotional needs and contexts.

“If the AI has the ability of a psychological counselor, it could comfort the user without fostering dependency, leaving when necessary and returning when needed.” (P6)

Realism in simulating the deceased's appearance, voice, and personality was viewed as critical for meaningful interaction.

“The AI resurrection of a loved one should simulate their appearance, voice, and personality as much as possible.” (P8)

Finally, participants highlighted the importance of interdisciplinary collaboration in achieving these design goals.

“Achieving the above design goals requires interdisciplinary collaboration, including the joint participation of technology, psychology, and related fields.” (P3)

#### 3.4.2 Expectations for appropriate use boundaries

Participants highlighted the importance of defining appropriate boundaries for griefbot use, encompassing issues of access, context, and patterns of use.

Regarding access, many participants suggested that griefbots should be restricted to close family members to prevent misuse.

“Only the deceased's family should be permitted to use this technology.” (P22)

However, views diverged regarding the extent of restriction. Some participants framed griefbots as a resource appropriate only under specific conditions or for particular groups.

“AI resurrection should function like a medical device, available only to specific groups, as each family's experience of loss differs.” (P8)

In contrast, others emphasized individual autonomy in decision-making.

“The decision should lie with the user, not others.” (P1)

“It shouldn’t be limited to those struggling with grief; everyone should have the right to use it.” (P2)

Participants also discussed appropriate contexts of use, favoring flexible, user-friendly environments over institutional settings.

“Not everyone wants to go to a hospital… this technology should be easy to use in a more convenient setting.” (P10)

Divergent views were also expressed regarding regulation of usage frequency and duration. While some participants emphasized self-regulation, others advocated structured oversight, particularly for vulnerable individuals.

“If the family member is within their control, they will manage how often they use the AI themselves.” (P6)

“Regulations should govern how often, how long, and where the technology is used.” (P3)

Some participants further suggested differentiated management strategies and time-limited use.

“Users should be categorized: those with self-control can use it freely, while others may require monitoring.” (P9)

“AI resurrection should help people accept loss, then decrease and stop, encouraging a return to life without the deceased.” (P14)

#### 3.4.3 Expectations for governance and regulatory oversight

Participants emphasized the important role of government in addressing potential risks associated with griefbot use. In particular, they expressed expectations regarding the need for public oversight to ensure safety, transparency, and ethical use, especially in response to concerns about fraud, misuse, and insufficient regulation.

##### 3.4.3.1 Government roles in building trust and preventing exploitation

Many participants suggested that government involvement could enhance trust and reduce the risk of exploitation. Public education and accessible service provision were identified as key strategies to mitigate fraud and misinformation.

“The government should also launch a major publicity campaign to educate people that this is a scientific technology, not a superstition. If the government provides the service at a low cost or for free, it may reduce the number of people being scammed.” (P3)

Government-led provision was also perceived as increasing users' sense of security, particularly in relation to privacy concerns.

“I believe that the regulation of AI resurrection technology should not only rely on private companies; it should be led by the government. This would make me feel safer, especially when it comes to privacy issues.” (P7)

##### 3.4.3.2 Government roles in regulation and enforcement

Participants further emphasized the need for formal regulatory frameworks to govern the development and use of griefbot technology. These expectations focused on establishing clear standards, defining acceptable boundaries, and ensuring accountability for misuse.

“As a product, it should have corresponding standards to regulate it. This way, we can better control the development of this new thing and avoid potential harm to society and humanity.” (P6)

“The government should intervene to regulate the use of this technology and impose legal punishments on those who misuse it.” (P19)

#### 3.4.4 Institutional and legal safeguards for griefbot use

Participants further articulated specific expectations regarding legal and institutional safeguards to address risks associated with griefbot use. These expectations were closely related to concerns about privacy violations, data misuse, and lack of consent identified in earlier themes.

##### 3.4.4.1 Legal safeguards for data, privacy, and content boundaries

Participants highlighted the need for clear legal frameworks to protect privacy, dignity, and data rights in the use of griefbot technology. Concerns were raised regarding the misuse of personal data and the ethical implications of reproducing sensitive content.

“AI-based resurrection could infringe on privacy or harm the dignity of the deceased. Rules and restrictions are essential to ensure ethical and legal use.” (P14)

Participants also emphasized the importance of defining data ownership and management.

“Data rights must be clearly defined, including whether data is deleted post-use or retained by the user.” (P12)

In addition, some participants noted that certain types of reconstructed content—particularly intimate or private material—may require explicit boundaries to ensure ethical use (P13).

##### 3.4.4.2 Professional standards and accountability mechanisms

Participants emphasized the importance of establishing professional standards and accountability mechanisms for individuals involved in the development and provision of griefbot services.

“It may be necessary to implement a certification process for practitioners to ensure they have the requisite professional competence.” (P5)

“Practitioners need specialized qualifications and training to provide higher-quality services and safeguard confidentiality.” (P11)

These accounts reflected participants’ expectations that user protection should involve not only technological regulation but also oversight of service providers.

##### 3.4.4.3 Consent and pre-mortem decision-making mechanisms

Participants further emphasized the need to establish mechanisms for consent and pre-mortem decision-making to protect the autonomy and dignity of the deceased.

“Many people may lose the ability to make decisions near the end of their life. It would be prudent to make our choices while we are still healthy, deciding in advance whether we want this technology.” (P7)

“It would be best to offer an informed consent form… so they can make an informed choice.” (P10)

These accounts reflected participants’ expectations that advance consent should be incorporated into the governance of griefbot technology.

## 4 Discussion

This study shows that public responses to griefbots are conditional rather than binary. Participants did not simply accept or reject the technology. Instead, they evaluated it based on context, timing, and user characteristics. Acceptability depended on factors such as emotional state, stage of grief, and purpose of use. Taken together, these findings can be interpreted as pointing toward a contextual acceptability perspective, in which attitudes toward griefbots are shaped by situational and relational factors rather than fixed ethical positions. This interpretation is consistent with emerging research suggesting that the social acceptability of digital resurrection is context-dependent and influenced by factors such as consent, purpose of use, and user vulnerability (26–28).

A dual effect was evident in the data, whereby the same technological features were associated with both benefits and risks. Griefbots were perceived as providing comfort and maintaining symbolic connections with the deceased (18), while also potentially reinforcing attachment and blurring the boundary between memory and ongoing interaction (31), which may contribute to emotional dependency or prolonged grief (32). This pattern may be understood as reflecting a form of techno-ambivalence, in which the same affordances simultaneously enable emotional support and psychological risk. These findings suggest that griefbots are better understood as contextually contingent technologies, rather than as uniformly beneficial or harmful.

The importance of consent emerged as a recurring concern. Questions were raised regarding whether the deceased had agreed to digital reconstruction, aligning with prior research that identifies consent as a central condition for ethical legitimacy (26, 27). However, the findings suggest that ethical concerns extend beyond the issue of permission alone. Concerns also focused on how accurately the deceased is represented, including potential distortions of personality, values, and identity. This highlights the importance of representational fidelity as a distinct dimension of ethical evaluation. In this sense, authenticity is not solely a technical matter of accuracy or resemblance, but also an ethical issue related to dignity, identity, and representational legitimacy (33). Greater realism may foster emotional connection but may also generate discomfort when the representation appears close to the person without being convincingly “them” (34, 35). These findings collectively suggest that governance considerations may need to address both consent and the integrity of posthumous representation.

Evaluation of griefbots varied according to purpose of use. Commemorative applications were more readily accepted, whereas commercial uses raised greater concern (28). Therapeutic use appeared highly context-dependent. User vulnerability also emerged as a key consideration, particularly among individuals experiencing acute grief, who were perceived to be at increased risk of emotional dependency or harm (29, 36). At the same time, these concerns extended beyond individual experiences, reflecting an awareness that griefbots are embedded within broader social and institutional contexts (37, 38). Issues such as access, profit, and the potential for exploitation were also raised, highlighting that these technologies do not operate in a social vacuum but are shaped by structural factors such as digital inequality and trust in platforms (25, 39). Social norms of bereavement were also implicated, as ongoing interaction with the deceased could be perceived by others as excessive or socially unsettling. These considerations collectively suggest the potential value of differentiated governance approaches, in which regulatory strategies are adapted according to purpose of use, user vulnerability, and broader social context.

Concerns regarding data use and transparency were also evident. Questions were raised about how personal data are collected and utilized, alongside uncertainty regarding how accurately the system represents the deceased. These issues were closely linked to trust. Expectations for external oversight were also apparent, suggesting that market-based mechanisms alone may be insufficient (40). Taken together, these concerns may be organized into several interrelated governance domains, including data governance, representational transparency, and institutional oversight.

This interpretation suggests the need for coordinated approaches that address these domains in an integrated manner. As summarized in Table 4, these findings can be tentatively translated into a set of multi-level governance considerations, linking empirical patterns of public perception to differentiated regulatory, platform-level, and professional responses.

**Table 4:** Mapping of empirical findings to governance implications under a contextual acceptability framework.

Key empirical finding	Description (from this study)	Governance implication
Contextual acceptability (situated evaluation of griefbots)	Acceptance is contingent on context, timing, user characteristics, and conditions of use (e.g., vulnerability, stage of grief)	Develop context-sensitive governance frameworks; avoid one-size-fits-all regulation; implement conditional access and adaptive use policies
Techno-ambivalence of affordances	The same affordances (e.g., simulated presence) generate both emotional comfort and potential harm (dependency, prolonged grief)	Introduce risk-tiering mechanisms and phase-specific use guidelines (e.g., early vs. later bereavement), with adaptive safeguards
Posthumous consent and legitimacy	Concerns regarding whether the deceased consented to digital reconstruction	Establish posthumous consent mechanisms, including advance directives, proxy authorization hierarchies, and auditable consent records
Representation fidelity and identity integrity	Risk of misrepresenting the deceased's personality, values, and intentions; concerns about dignity and authenticity	Define standards for representational fidelity; require disclosure of training data sources, system limitations, and generative uncertainty
Purpose-dependent evaluation	Differential acceptance across commemorative, therapeutic, and commercial uses	Implement purpose-differentiated governance; stricter controls for commercial monetization; require clinical oversight in therapeutic contexts
User vulnerability and emotional dependency	Heightened risk among individuals in acute grief, including overuse and attachment intensification	Introduce user protection safeguards, such as usage limits, nudges, cooldown periods, and clinician-in-the-loop mechanisms for high-risk users
Data governance and transparency concerns	Concerns about data misuse, unclear provenance, and opacity in generated outputs	Strengthen data governance frameworks, including data minimization, provenance tracking, explainability, and user control over data use
Expectation of oversight and accountability	Participants expect external regulation beyond market self-governance	Establish formal regulatory and professional oversight mechanisms, including auditing, liability frameworks, and compliance standards
Integrative governance architecture (cross-cutting)	Findings collectively reflect multi-dimensional ethical tensions (acceptability, ambivalence, representation, vulnerability)	Require multi-layered governance architecture integrating data governance, user protection, and institutional oversight across system lifecycle

This study contributes by clarifying the structure of public perceptions of griefbots. Acceptance appears to be conditional and relational, shaped by multiple interacting factors rather than a single principle. By outlining a contextual acceptability perspective and identifying key dimensions such as techno-ambivalence and governance relevance, this study provides an empirically grounded basis for understanding how AI-mediated mourning technologies may be evaluated in practice. In this sense, it complements existing normative and institutional research by offering insight into how governance-relevant concerns are articulated in real-world contexts.

## 5 Limitation

Several limitations should be noted. First, this study explored public perceptions of griefbots rather than actual long-term experiences of use. Participants' accounts were therefore shaped by anticipation, imagination, and moral reflection in response to a still-emerging technology, rather than by sustained interaction with clearly defined existing systems. Second, the findings are situated within a specific cultural and social context. Attitudes toward death, bereavement, family obligation, and digital afterlife practices may differ across settings. Third, although participants varied in age and background, this study was not designed to compare responses systematically across different bereavement histories or user groups. Future research could therefore examine how griefbot perceptions vary across cultural contexts, types of loss, and levels of bereavement vulnerability. It would also be valuable to study actual use experiences over time, especially in relation to emotional adaptation, authenticity judgments, and the role of external support, clinical guidance, or regulatory safeguards.

## 6 Conclusion

Griefbots were perceived neither as inherently beneficial nor inherently harmful. Rather, they were understood as technologies whose comfort and risk stem from the same capacity to simulate continued presence after death. Public evaluation was shaped not only by perceived utility, but also by deeper questions about authenticity, mourning, personhood, and the moral status of posthumous representation. Acceptability therefore emerged as conditional rather than fixed, shaped by when, for whom, and under what conditions griefbots might be used. Recognizing this conditional and negotiated structure of acceptability is essential for any responsible attempt to develop, regulate, or integrate griefbots into bereavement-related contexts.

## Data Availability

The datasets presented in this article are not readily available because they contain qualitative interview data that may compromise participant confidentiality. Requests to access the datasets should be directed to the corresponding author.

## References

[1] Haugan G Jacobsen FF Søvde BE Drageset J. We are the ones closest to ours and can see what they may need”: family involvement in nursing homes: relief and safety versus burden, worries and frustration. Geriatr Nurs. (2025) 66:103580. 10.1016/j.gerinurse.2025.103580 40840157

[2] Simon NM Shear MK. Prolonged grief disorder. N Engl J Med. (2024) 391(13):1227–36. 10.1056/NEJMcp2308707 39589372

[3] Lundorff M Holmgren H Zachariae R Farver-Vestergaard I O’Connor M. Prevalence of prolonged grief disorder in adult bereavement: a systematic review and meta-analysis. J Affect Disord. (2017) 212:138–49. 10.1016/j.jad.2017.01.030 28167398

[4] van Mol MMC Wagener S Latour JM Boelen PA Spronk PE den Uil CA. Developing and testing a nurse-led intervention to support bereavement in relatives in the intensive care (BRIC study): a protocol of a pre-post intervention study. BMC Palliat Care. (2020) 19(1):130. 10.1186/s12904-020-00636-8 32811499 PMC7433274

[5] Cambonie G Desage C Thaller P Lemaitre A de Balanda KB Combes C. Context of a neonatal death affects parental perception of end-of-life care, anxiety and depression in the first year of bereavement. BMC Palliat Care. (2023) 22(1):58. 10.1186/s12904-023-01183-8 37173678 PMC10182590

[6] Djelantik AAAMJ Smid GE Mroz A Kleber RJ Boelen PA. The prevalence of prolonged grief disorder in bereaved individuals following unnatural losses: systematic review and meta-regression analysis. J Affect Disord. (2020) 265:146–56. 10.1016/j.jad.2020.01.034 32090736

[7] Hilberdink CE Ghainder K Dubanchet A Hinton D Djelantik AMJ Hall BJ. Bereavement issues and prolonged grief disorder: a global perspective. Camb Prisms Glob Ment Health. (2023) 10:e32. 10.1017/gmh.2023.28 PMC10579660 37854407

[8] Stroebe M Stroebe W Schut H Boerner K. Grief is not a disease but bereavement merits medical awareness. Lancet. (2017) 389(10067):347–9. 10.1016/S0140-6736(17)30189-7 28137681

[9] Öhman C Floridi L. An ethical framework for the digital afterlife industry. Nat Hum Behav. (2018) 2(5):318–20. 10.1038/s41562-018-0335-2 30962597

[10] Hadlington L Karanika-Murray M Slater J Binder J Gardner S Knight S. Public perceptions of the use of artificial intelligence in defence: a qualitative exploration. AI Soc. (2025) 40(2):277–90. 10.1007/s00146-024-01871-w

[11] van der Goot MJ Koubayová N van Reijmersdal EA. Understanding users’ responses to disclosed vs. Undisclosed customer service chatbots: a mixed methods study. AI Soc. (2024) 39(6):2947–60. 10.1007/s00146-023-01818-7

[12] Liu YC. The rise, types and social impacts of artificial intelligence “resurrection” technology. Renmin Luntan. (2024) (11):46–50.

[13] Dautenhahn K. Socially intelligent robots: dimensions of human-robot interaction. Philos Trans R Soc Lond B Biol Sci. (2007) 362(1480):679–704. 10.1098/rstb.2006.2004 17301026 PMC2346526

[14] Abbasi J. Socially assistive robots help patients make behavioral changes. JAMA. (2017) 317(24):2472–4. 10.1001/jama.2017.5682 28593246

[15] Nichol B McCready J Erfani G Comparcini D Simonetti V Cicolini G. Exploring the impact of socially assistive robots on health and wellbeing across the lifespan: an umbrella review and meta-analysis. Int J Nurs Stud. (2024) 153:104730. 10.1016/j.ijnurstu.2024.104730 38430662

[16] Tapus A Mataric MJ Scassellati B. Socially assistive robotics. IEEE Robot Autom Mag. (2007) 14(1):35–42. 10.1109/MRA.2007.339605

[17] Wright C. Should we communicate with the dead to assuage our grief? An ubuntu perspective on using griefbots. Data Policy. (2024) 6:e55. 10.1017/dap.2024.61

[18] Jiménez-Alonso B Brescó De Luna I. Griefbots: a new way of communicating with the dead? Integr Psychol Behav Sci. (2023) 57(2):466–81. 10.1007/s12124-022-09679-3 35293606

[19] Lindemann NF. The ethics of “deathbots”. Sci Eng Ethics. (2022) 28(6):60. 10.1007/s11948-022-00417-x 36417022 PMC9684218

[20] Pizzoli SF Vergani L Monzani D Scotto L Cincidda C Pravettoni G. The sound of grief: a critical discussion on the experience of creating and listening to the digitally reproduced voice of the deceased. Omega (Westport). (2024) 92(4):2218–2226. 10.1177/00302228231225273 38176688 PMC12891239

[21] Duffy C. ‘There are no guardrails’: this mom believes an AI chatbot is responsible for her son’s suicide. CNN [Internet] (2024). Available online at: https://www.cnn.com/2024/10/30/tech/teen-suicide-character-ai-lawsuit/index.html (Accessed 2024 December 24).

[22] McCradden M Hui K Buchman DZ. Evidence, ethics and the promise of artificial intelligence in psychiatry. J Med Ethics. (2023) 49(8):573–9. 10.1136/jme-2022-108447 36581457 PMC10423547

[23] Zhang LH. Ethical challenges of artificial intelligence “resurrection” technology applications. Renmin Luntan. (2024) (11):55–8.

[24] Bao A Zeng Y. Artificial intelligence risks, attention allocation and priorities. J Med Ethics. (2024) 50(12):822–3. 10.1136/jme-2024-110279 39209375

[25] Sheir S Manzini A Smith H Ives J. Adaptable robots, ethics, and trust: a qualitative and philosophical exploration of the individual experience of trustworthy AI. AI Soc. (2025) 40(3):1735–48. 10.1007/s00146-024-01938-8 40225305 PMC11985637

[26] Iwasaki M. Digital clones of the deceased in mental health care: promises and perils. Digit Health. (2025) 11:20552076251332839. 10.1177/20552076251332839 40297348 PMC12034995

[27] Iwasaki M. Digital cloning of the dead: exploring the optimal default rule. Asian J Law Econ. (2024) 15(1):1–29. 10.1515/ajle-2023-0125

[28] Arbel YA Lopez A. Governing generative AI beyond the grave. SSRN [Preprint] (2026). Available online at: https://papers.ssrn.com/sol3/papers.cfm?abstract_id=5380233 (Accessed May 2, 2026).

[29] Yang YT. Digital resurrection and posthumous identity: toward a cross-cultural neurorights framework. AJOB Neurosci. (2025) :1–16. 10.1080/21507740.2025.2519440 40554775

[30] Colaizzi PF. Psychological research as the phenomenologist views it. In: Valle RS King M, editors. Existential-phenomenological Alternatives for Psychology. New York: Oxford University Press (1978). p. 48–71.

[31] Voinea C. On grief and griefbots. Think. (2024) 23(67):47–51. 10.1017/S1477175623000490

[32] Bao A Zeng Y. Embracing grief in the age of deathbots: a temporary tool, not a permanent solution. Ethics Inf Technol. (2024) 26(1):7. 10.1007/s10676-024-09744-y

[33] Morse T. Digital necromancy: users’ perceptions of digital afterlife and posthumous communication technologies. Inf Commun Soc. (2024) 27(2):240–56. 10.1080/1369118X.2023.2205467

[34] Moore RK. A Bayesian explanation of the “uncanny valley” effect and related psychological phenomena. Sci Rep. (2012) 2:864. 10.1038/srep00864 23162690 PMC3499759

[35] Zhang YL. Interaction design of digital human parents for left-behind children and auxiliary parenting app design (Master’s thesis). Beijing University of Posts and Telecommunications, Beijing (2023).

[36] Lemma A. Mourning, melancholia and machines: an applied psychoanalytic investigation of mourning in the age of griefbots. Int J Psychoanal. (2024) 105(4):542–63. 10.1080/00207578.2024.2342917 38738382

[37] Smoła P Młoźniak I Wojcieszko M Zwierczyk U Kobryn M Rzepecka E. Attitudes toward artificial intelligence and robots in healthcare in the general population: a qualitative study. Front Digit Health. (2025) 7:1458685. 10.3389/fdgth.2025.1458685 39931116 PMC11808042

[38] Gocmen Baykara Z Keles S Demir Karabulut S Gul S Eren H Durmus Iskender M. The effect of professional education on medical and nursing students’ attitudes toward death. Death Stud. (2022) 46(7):1728–40. 10.1080/07481187.2020.1850546 33287666

[39] McStay A. The hidden influence: exploring presence in human-synthetic interactions through ghostbots. Ethics Inf Technol. (2024) 26(3):48. 10.1007/s10676-024-09786-2

[40] Harbinja E Edwards L McVey M. Governing ghostbots. Comput Law Secur Rev. (2023) 48:105791. 10.1016/j.clsr.2023.105791

